# Global and local missions of cAMP signaling in neural plasticity, learning, and memory

**DOI:** 10.3389/fphar.2015.00161

**Published:** 2015-08-04

**Authors:** Daewoo Lee

**Affiliations:** Neuroscience Program, Department of Biological Sciences, Ohio University, Athens, OH, USA

**Keywords:** *Drosophila melanogaster*, synaptic plasticity, associative learning and memory, rutabaga, dunce, cAMP homeostasis

## Abstract

The fruit fly *Drosophila melanogaster* has been a popular model to study cAMP signaling and resultant behaviors due to its powerful genetic approaches. All molecular components (AC, PDE, PKA, CREB, etc) essential for cAMP signaling have been identified in the fly. Among them, adenylyl cyclase (AC) gene *rutabaga* and phosphodiesterase (PDE) gene *dunce* have been intensively studied to understand the role of cAMP signaling. Interestingly, these two mutant genes were originally identified on the basis of associative learning deficits. This commentary summarizes findings on the role of cAMP in *Drosophila* neuronal excitability, synaptic plasticity and memory. It mainly focuses on two distinct mechanisms (global versus local) regulating excitatory and inhibitory synaptic plasticity related to cAMP homeostasis. This dual regulatory role of cAMP is to increase the strength of excitatory neural circuits on one hand, but to act locally on postsynaptic GABA receptors to decrease inhibitory synaptic plasticity on the other. Thus the action of cAMP could result in a global increase in the neural circuit excitability and memory. Implications of this cAMP signaling related to drug discovery for neural diseases are also described.

Since its discovery in 1958 (Sutherland, 1992; Pittenger et al., 2012), the cyclic AMP signaling pathway has been shown to regulate a plethora of cellular functions including energy metabolism, gene expression, development, apoptosis and exocytosis. This second messenger molecule is synthesized from ATP by a family of enzymes called adenylyl cyclases (ACs), activated by G-protein coupled receptors (GPCRs). Considering the importance of this intracellular signaling, it is not surprising that cAMP levels in the cell are negatively regulated by another group of enzymes—phosphodiesterases (PDEs). An increase in cAMP levels activates protein kinase A (PKA) which then phosphorylates target proteins including other kinases, transcriptional factors and ion channels. Therefore, its effects can be achieved by short- or long-term fashion. The latter is a protein synthesis-dependent process and thus mediated by a well-known transcription factor cAMP response element-binding protein (CREB).

Many isoforms of ACs and PDEs have been identified in the nervous system, indicating critical roles of cAMP in neural function (Pittenger et al., 2012). Indeed, cAMP signaling in the brain is known to mediate numerous neural processes from development, cellular excitability, synaptic plasticity, learning and memory, pain and motor function to neurodegeneration and drugs of abuse (Pierre et al., 2009; Bollen and Prickaerts, 2012; Kandel, 2012; Pittenger et al., 2012). In this commentary, I will specifically focus on the role of cAMP signaling in neural excitability, synaptic plasticity, learning and memory. Other important functions of cAMP signaling in the nervous system can be found in several outstanding reviews elsewhere (refer to Pittenger et al., 2012).

The cAMP signaling pathway mediates synaptic plasticity in both vertebrates and invertebrates. In a sea slug *Aplysia*, it was demonstrated that cAMP signaling mediates short- and long-term facilitation (LTF) at sensorimotor synapses (Brunelli et al., 1976; Schacher et al., 1988; Kaang et al., 1993; Bartsch et al., 1995; Kandel, 2012). The LTF was dependent on the action of CREB and new protein synthesis, and subsequently shown to mediate memory formation in *Aplysia*. In the rodent hippocampus, cAMP is involved in long-term potentiation (LTP) at excitatory glutamatergic synapses (Frey et al., 1993; Weisskopf et al., 1994; Silva et al., 1998). All these findings support the idea that cAMP-dependent synaptic plasticity is responsible for behavioral learning and memory at the whole organism level in both invertebrates and vertebrates. The fruit fly *Drosophila melanogaster* has been widely used to study molecular and cellular mechanisms of learning and memory due to its sophisticated genetic approaches. In addition, *Drosophila* nervous system contains all molecular components (e.g., AC, PDE, PKA, CREB, etc) essential for the cAMP signaling pathway. *Drosophila* AC gene *rutabaga* and PDE gene *dunce* have been intensively studied to understand the role of cAMP signaling in the nervous system. These two mutant genes were originally identified on the basis of associative learning deficits (Dudai et al., 1976; Chen et al., 1986; Levin et al., 1992; Busto et al., 2010). Further, the cAMP signaling pathway in the fly regulates synaptic plasticity at both peripheral neuromuscular junction (NMJ) as well as central synapses, where alterations in facilitation and post-tetanic potentiation were observed in mutant flies (*dunce* and *rutabaga*) with defects in cAMP signaling (Zhong and Wu, 1991; Cheung et al., 1999; Lee and O’Dowd, 2000; Ganguly and Lee, 2013). All these findings strongly support that *Drosophila* is an excellent model system to study the role of cAMP signaling in synaptic plasticity and the resultant behavior—learning and memory.

In this mini review, I summarize the findings on the role of cAMP in *Drosophila* neuronal excitability, synaptic plasticity, and learning and memory. The main emphasis is to understand distinct mechanisms (global versus local) regulating excitatory and inhibitory synaptic transmission related to cAMP homeostasis. At the end, I comment on implications of this research on disease therapy.

## Global Missions of cAMP Signaling

### Neuronal Excitability

One common effect of cAMP on neural function is to modulate cellular excitability. In an *Aplysia* sensory neuron, cAMP increased spike duration and excitability (Goldsmith and Abrams, 1992). Excitability of the rodent hippocampal neurons was also increased by cAMP signaling which subsequently enhances LTP (Gruart et al., 2012). Further, striatal neuronal excitability has been shown to be regulated by cAMP signaling (Threlfell and West, 2013).

In *Drosophila* NMJ, the excitability of a motor neuron is regulated by cAMP signaling (Zhong and Wu, 1991, 2004). This change increases the excitability of presynaptic terminals, thus influencing release of neurotransmitter (NT) glutamate. This cAMP effect on excitability was also observed in *Drosophila* central nervous system (CNS). In contrast to mammalian CNS, acetylcholine is the primary excitatory NT in *Drosophila* CNS (Restifo and White, 1990; Lee and O’Dowd, 1999). These cholinergic neurons play a critical role in almost all higher brain function in *Drosophila* as glutamatergic neurons do in mammalian CNS. Due to difficulties in recording electrical signals from a single neuron in the fly brain, *Drosophila* primary neuronal culture has become a good alternative to study ionic and synaptic currents from central neurons (O’Dowd, 1995; Lee and O’Dowd, 1999; Ganguly and Lee, 2013). When fly cholinergic neurons were focally exposed to a popular AC activator forskolin (FSK), action potential (AP) frequency was drastically increased. GABAergic neurons also showed an increase in AP in response to the focal application of FSK (Ganguly and Lee, 2013). This change is a direct effect of cAMP as excitatory cholinergic inputs to GABAergic neurons were blocked by an acetylcholine receptor (AChR) blocker curare. In *Drosophila*, live cholinergic and GABAergic neurons can be easily identified using a live fluorescent marker (e.g., RFP or GFP; Wiemerslage et al., 2013). Increased excitability by cAMP is primarily achieved through PKA, which mediates a phosphorylation-induced reduction in potassium channel conductance (Wright and Zhong, 1995; Delgado et al., 1998). Taken together, all these findings show that cAMP-PKA signaling directly enhanced the excitability of all types of neurons—not only inhibitory GABAergic (Ganguly and Lee, 2013) but also excitatory cholinergic and glutamatergic neurons (Zhong and Wu, 1991, 2004; Lee and O’Dowd, 2000). Further, these findings indicate that cAMP signaling increases pre-synaptic NT release through enhanced excitability.

### Synaptic Plasticity in CNS

Synaptic transmission is a primary way to communicate between neurons in the brain. The synapse is not static, rather dynamically changes its strength which is known as synaptic plasticity, an important subcellular mechanism underlying learning and memory (Bhalla and Iyengar, 1999; Kandel, 2001). A key molecule involved in this plasticity is the second messenger cAMP.

**FIGURE 1:**
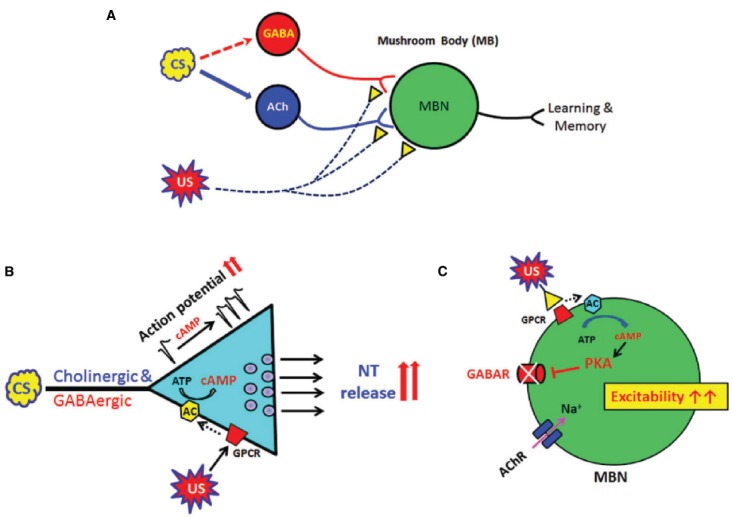
**Global and local actions of cAMP signaling in neural plasticity, learning and memory in ***Drosophila***. (A)** A diagram showing *Drosophila* neural circuits which mediate associative learning and memory formation. Sensory information (CS) is mainly transduced through excitatory cholinergic synaptic inputs to the fly learning and memory center, mushroom body (MB). Inhibitory GABAergic inputs to MB are known to shape this sensory information transduction although its sensory processing circuits remain to be explored (dotted arrow). US (reward or punishment) is mediated through modulatory synaptic inputs such as dopaminergic or serotonergic. Association of the CS and US in MB is the basis of *Drosophila* learning. **(B)** Cyclic AMP has global (or general) roles to increase presynaptic excitability and neurotransmitter release. These changes are found in all central neurons including excitatory cholinergic and inhibitory GABAergic neurons. **(C)** In contrast, cAMP suppresses ionotropic GABA receptors in the postsynaptic neuron and thus enhances overall excitability in MB neural circuits. Acetylcholine receptors (AChRs) in *Drosophila* postsynaptic neurons were not affected by cAMP signaling (Lee and O’Dowd, 2000). Conditional stimulus (CS); MB neurons (MBN); unconditional stimulus (US).

The cAMP-PKA pathway is known to regulate synaptic plasticity (e.g., LTP) in the mossy fibers and CA3 pyramidal cells in hippocampal slices (Nicoll and Malenka, 1995; Silva et al., 1998). The cAMP signaling pathway has been shown to increase the pre-synaptic vesicle release probability (Chen and Regehr, 1997), through enhanced vesicle docking before exocytosis (Sudhof, 2004). In *Aplysia*, PKA increases an influx of Ca^2+^ into the pre-synaptic neuron, facilitates vesicle fusion, and glutamate release resulting in short term facilitation by inhibiting the S-type K^+^ channels (Kandel, 2001). Studies in *Drosophila* NMJ have shown that cAMP alters pre-synaptic release probability and facilitation (Zhong and Wu, 1991), which can be achieved by the reduction of K^+^ currents (Zhong et al., 1992) or by activation of hyperpolarization-activated cyclic nucleotide-gated (HCN) channels (Cheung et al., 2005). We have demonstrated that cAMP signaling can regulate functional plasticity, independent of differentiation, at excitatory cholinergic synapses between cultured *Drosophila* neurons (Lee and O’Dowd, 2000). Presynaptic GABA release was also greatly increased by an AC activator FSK (Ganguly and Lee, 2013). Further, it has been shown that facilitation is impaired in cAMP signaling mutants (Zhao and Wu, 1997; Lee and O’Dowd, 2000; Ganguly and Lee, 2013). All the findings show that the cAMP-PKA pathway plays an important role in regulating plasticity at excitatory as well as inhibitory synapses in *Drosophila* CNS. Therefore, global actions of cAMP signaling on synaptic plasticity appear to be enhanced presynaptic release of NT via increasing presynaptic excitability in all types of neurons including excitatory cholinergic and inhibitory GABAergic neurons (Figure 1).

## Local Mission of cAMP Signaling

Cyclic AMP-PKA signaling generally (and globally) increases neuronal excitability and presynaptic release of NTs in *Drosophila* nervous system. This increase was observed not only at excitatory but also inhibitory synapses. This can be contradictory because an increase in inhibitory synaptic transmission by cAMP is expected to reduce overall strength of excitatory synaptic transmission in certain neural circuits. Therefore, we further examined the role of cAMP signaling in inhibitory GABAergic synaptic transmission in *Drosophila* (Ganguly and Lee, 2013). When postsynaptic cAMP signaling was specifically blocked by a membrane-permeable PKA inhibitor, the frequency of GABAergic synaptic currents was increased by focal application of FSK like excitatory cholinergic synaptic currents (see above). However, without the blocker in a postsynaptic neuron, GABAergic synaptic transmission was suppressed by FSK even if presynaptic GABA release was increased (Ganguly and Lee, 2013). This confirmed that PKA suppresses GABAergic synaptic transmission by regulating postsynaptic GABA receptor sensitivity through phosphorylation. Among three *Drosophila* ionotropic GABA receptors (RDL, LCCH3, and GRD; Harvey et al., 1994; Hosie et al., 1997), the GABA *resistant to dieldrin* (RDL) receptor subunit is widely expressed in several regions of the *Drosophila* brain (Harrison et al., 1996) and its expression in the fly learning and memory center mushroom body (MB) is inversely correlated to olfactory learning (Liu et al., 2007). Based on the observation that RDL containing GABA receptors mediate the majority of GABAergic synaptic currents in *Drosophila* (Lee et al., 2003; Ganguly and Lee, 2013), the action of cAMP on GABAergic synaptic currents is likely through the GABA RDL subunit. PKA-mediated phosphorylation of RDL subunits and subsequent GABA receptor internalization may occur specifically (and locally) in the postsynaptic region (Mou et al., 2011; Vithlani et al., 2011).

*Drosophila* brain has shown the presence of a large number of inhibitory GABAergic interneurons, some of which innervate into the MB (Yasuyama et al., 2002; Busto et al., 2010). These interneurons have been shown to be important for various forms of information processing and behaviors including learning and memory (Olsen and Wilson, 2008; Isaacson and Scanziani, 2011; Wilson, 2013). Strengthening in the efficacy of excitatory transmission causes enhanced neural circuit plasticity. Therefore, the suppression of inhibitory transmission by a common second messenger like cAMP is expected to further increase the neural circuit excitability. In *Drosophila*, cAMP-PKA signaling increases excitability at the cholinergic synapses (Yuan and Lee, 2007) but decreases the conductance of postsynaptic GABA receptors (Ganguly and Lee, 2013). These findings demonstrate a novel dual regulatory role of cAMP by showing that it increases overall presynaptic function globally, but acts locally on postsynaptic GABA receptors to decrease GABAergic plasticity. Thus the action of cAMP results in further increases in neural excitability (Figure 1).

## Cyclic AMP Homeostasis, Neural Circuits, Learning, and Memory

A number of *Drosophila* mutants showing defects in the cAMP-signaling cascade were originally identified on the basis of associative learning deficits (Dudai et al., 1976; Livingstone et al., 1984; Chen et al., 1986; Levin et al., 1992). Among them, a *Drosophila* Ca^2+^/CaM-dependent AC *rutabaga* is known to function as a coincidence detector during learning and memory consolidation (Tomchik and Davis, 2009; Gervasi et al., 2010). A typical associative learning task is comprised of two different stimuli—conditional (CS; sensory input such as smell or visual) and unconditional stimuli (US; reward or punishment). In *Drosophila*, excitatory cholinergic and GABAergic inputs are considered to deliver CS signals while a variety of synaptic modulators (e.g., dopamine, serotonin, etc) are involved in the transduction of US signals (Figure 1). The US modulators typically stimulate GPCR to activate AC while synaptic inputs from CS neural circuits increase cellular excitability and also Ca^2+^/CaM. Therefore, AC serves as a coincidence detector of CS and US. A *Drosophila* PDE4 homolog *dunce* is also known to cause defects in short-term memory (Dudai et al., 1976; Chen et al., 1986). Both *rutabaga* and *dunce* enzymes in *Drosophila* show changes in the strength of *Drosophila* excitatory synapses (Zhong and Wu, 1991; Lee and O’Dowd, 2000) as well as inhibitory GABAergic synapses (Ganguly and Lee, 2013). Therefore, cAMP-dependent synaptic plasticity must be an essential feature for neural circuits in mediating learning and memory.

Interestingly, consequences of *rutabaga* and *dunce* mutants are opposite in terms of intracellular cAMP levels. The former decreases basal cAMP levels and thus does not able to temporally increase cAMP in response to acute stimulation, while the letter increases the basal level of intracellular cAMP much more than that in wild type. However, both mutations cause defects in synaptic plasticity and memory, strongly indicating the importance of cAMP homeostasis. Proper regulation of intracellular cAMP appears to be critical for neural plasticity and memory in fly. Thus it is of interest to understand the role of these two enzymes maintaining homeostasis of cAMP in *Drosophila* neurons.

The *Drosophila* MB is comprised of several subdivisions (e.g., α, β, γ lobe, etc) (Keene and Waddell, 2007; Guven-Ozkan and Davis, 2014). Different MB neurons are involved in distinct types and/or phases of associative learning and memory in the fly. Further details can be found in several excellent reviews on MB structure and function (Fahrbach, 2006; Waddell, 2013; Guven-Ozkan and Davis, 2014). The synaptic inputs to MB are mainly coming from excitatory cholinergic and GABAergic neurons from the centers of sensory system (e.g., antennal lobes for olfaction) as they primarily mediate sensory information. For example, projection neurons (PNs) in antennal lobes relay sensory signals to MB neurons for olfactory associative learning. PN neurons are cholinergic and thus excitatory synaptic inputs. MB neurons also receive synaptic inputs from GABAergic neurons mainly from the region called lateral horn (LH; Busto et al., 2010). Since *rutabaga* and *dunce* are preferentially expressed in MB, cAMP signaling is important for synaptic plasticity in MB neurons and also essential for learning and memory.

Learning and memory defects in *rutabaga* can be easily explained as its basal cAMP level is too low so that no neural input signal can induce proper cAMP-dependent synaptic plasticity mediating learning memory. Interestingly, *dunce* mutants with high levels of cAMP in MB neurons also show defects in short-term memory (Gervasi et al., 2010). Further, the *dunce* MB neurons show an increase in PKA levels. These findings suggest that cAMP-mediated potentiation of cholinergic synaptic transmission and inhibition of GABA receptor should be greater in *dunce* neurons. However, the *dunce* and *rutabaga* mutants, despite having opposing effects on cellular cAMP levels, showed very similar defects in synaptic plasticity at both excitatory and inhibitory synapses (Lee and O’Dowd, 2000; Ganguly and Lee, 2013). Several other studies have also shown that *dunce* and *rutabaga* have similar defects in growth cone motility, neural plasticity and more importantly, short-term memory (Kim and Wu, 1996; Gasque et al., 2006). Although the effects of cAMP on cholinergic and GABAergic synaptic plasticity in *dunce* and *rutabaga* mutants are similar, it is very likely that the molecular mechanisms underlying these responses differ in the two mutants. It has been shown that increased PKA activity in mouse hippocampus hyper-phosphorylates several downstream molecular targets including a tyrosine phosphatase, correlates with decreased PDE protein levels and results in memory defects (Giralt et al., 2011). Therefore, it is expected that high basal levels of cAMP due to the *dunce* mutation leads to the activation of phosphatase(s) and thus reduces the effects of cAMP. However, this remains to be explored in *Drosophila* nervous system. Taken together, all these findings strongly suggest that disruption of cellular cAMP homeostasis can alter excitatory cholinergic and inhibitory GABAergic synaptic plasticity and hence cause defects in associative learning, although the underlying mechanisms leading to this effect can be different (e.g., reduced PKA activity in *rutabaga* versus increased phosphatase activity in *dunce*).

## Drug Targets in cAMP Signaling

Cyclic AMP is the most abundant and important second messenger in the nervous system. Therefore, it makes sense that its signaling involves a variety of physiological and pathological processes such as learning and memory, pain, drug addiction and neurodegeneration. Molecular components in cAMP-PKA signaling pathway should be excellent targets for the development of new therapeutic strategies. These components in cAMP signaling are very well conserved throughout animal groups and, therefore, findings from *Drosophila* can be directly implicated in mammalian systems including human.

Memory is an important physiological process for survival and better quality of life. Thus any compromise in this behavior is likely a problem as seen in Alzheimer’s disease (AD) and mental retardation. Given the importance of cAMP signaling in neural plasticity and cognition, any molecular component in this signaling pathway can be a potential target for drug development to enhance cognition. Particularly two enzymes have drawn more attention. The first target is a PDE which down-regulates cAMP-PKA signaling. Therefore, PDE inhibitors will prolong cAMP signaling and produce higher levels potentially enhancing cognition. Indeed, a PDE4 inhibitor HT-0712 has been shown to improve hippocampus-mediated memory in mice (Peters et al., 2014). HT-0712 also increases CRE-mediated gene expression and ameliorates spatial memory impairment in aged mice, therefore, it could be used to treat age-associated memory impairment (AAMI) in humans. In fact, clinical studies with this drug have been carried out and show significant effects on long-term memory in AAMI patients (refer to: www.dartneuroscience.com). Since an increase of cAMP-specific PDE mRNAs was observed in early stages of AD (Bollen and Prickaerts, 2012), PDE inhibitors can be used to slow/treat this disease. However, it should be kept in mind that the chronic increase of basal cAMP by PDE inhibitors can give negative impacts on learning and memory as seen in the *dunce* mutants.

The second promising drug target for cAMP signaling is AC. AC1/AC8 double knockout mice showed loss of LTP as well as memory (Wong et al., 1999). Further, beta amyloid peptides interfere with AC-dependent LTP in hippocampus (Yamamoto et al., 1997). Findings with *rutabaga* fly mutants are consistent as these flies showed defects in synaptic plasticity and short-term memory. Therefore, drugs that stimulate AC activities can be useful to treat memory deficits although no drug is on a clinical test yet. AC is an attractive drug target to be further explored.

Additional potential targets in the cAMP signaling pathway are PKA and CREB-binding protein (CBP). Rubinstein-Taybi syndrome (RTS), a genetic disorder showing mental retardation and physical abnormalities (Bourtchouladze et al., 2003) is known to be caused by mutations of CBP. Regarding the role of CREB and CBP in the nervous system, it is noteworthy to mention that some addictive drugs (e.g., amphetamine, opiates) also alter CREB expression in several brain areas (e.g., nucleus accumbens and hippocampus; Robbins et al., 2007; Nestler, 2013) indicating involvement of cAMP signaling in drug addiction. Interestingly, *Drosophila* has been used as a favorable model animal to study actions of addictive drugs such as cocaine and alcohol (Kaun et al., 2012). A recent study showed that *Drosophila rutabaga* in MB neurons is necessary for robust ethanol self-administration (Xu et al., 2012) demonstrating the role of cAMP signaling in reinforced behaviors. Therefore, PKA and CREB-related proteins can be excellent drug targets to treat not only cognitive deficits but also drug addiction.

Given the suitability of multiple drug targets in cAMP signaling, one important question is the therapeutic safety in addition to drug potency. In other words, how can we deliver a drug to a specific target in order to minimize toxic side effects? Chemogenetic tools have been developed to enhance specific drug delivery spatially and temporally (Sternson and Roth, 2014). One such tool is designer receptors exclusively activated by designer drugs (DREADDs), which can increase or decrease intracellular cAMP levels depending on the receptor type by a biologically inert chemical (Becnel et al., 2013). Using this tool, cAMP signaling can be regulated in a specific set of neurons (e.g., hippocampus, MB) as well as duration of action (e.g., acute versus chronic). Therefore, CREB-mediated long-term effects can be also induced. In addition, DREADD can be an excellent research tool to uncover more specific roles of cAMP signaling in a variety of neuronal processes including learning and memory.

## Conclusion

In this commentary, I focused on the role of cAMP signaling in neural excitability, synaptic plasticity, learning and memory. On the basis of work from *Drosophila*, dual regulatory roles (global versus local) of cAMP signaling are to increase the strength of excitatory neural circuits on one hand, but to act locally on postsynaptic GABA receptors to decrease inhibitory synaptic plasticity on the other. Thus the action of cAMP could result in a global increase in the neural circuit excitability and memory. The cAMP signaling is also implicated in pain processing, AD and drug addiction. All these normal and disease-related behaviors are mediated through interaction between specific neural circuits comprised of excitatory and inhibitory synapses. Therefore, knowledge gained from the studies of cAMP signaling can contribute to the development of new or more effective drugs. Since molecular components and functions of cAMP signaling pathway have been well conserved in *Drosophila*, it could be a useful animal model to study mechanisms underlying behaviors mediated by cAMP signaling at the molecular, physiological and circuit levels. The fly can also be an excellent drug discovery platform for diseases with defects in cAMP signaling.

### Conflict of Interest Statement

The author declares that the research was conducted in the absence of any commercial or financial relationships that could be construed as a potential conflict of interest.
